# Preparation of Novel Nanomaterial and Its Application in Food Industry

**DOI:** 10.3390/foods11101382

**Published:** 2022-05-11

**Authors:** Hong Wu, Hui Zhang

**Affiliations:** 1 School of Food Science and Engineering, South China University of Technology, Guangzhou 510000, China; 2 College of Biosystems Engineering and Food Science, Zhejiang University, Hangzhou 310058, China

Nanotechnology has offered a wide range of opportunities for the development and application of structures, materials, or systems with new properties in the food industry in recent years. The developed nanomaterials could greatly improve not only food quality and safety but also the foods’ health benefits. In this special issue, different nano-sized vehicles (e.g., nanoparticle, nanoliposome, nanofiber, nanobody) are reported as efficient bioactives delivery systems and sensitive detection materials.

To resolve the low chemical instability, poor water solubility, and intestinal efflux limitations and challenges of bioactive ingredients, constructing an effective delivery vehicle using food-grade polymers is supposed to be a novel and feasible strategy. For example, Chen et al. [1] encapsulated hydrophobic naringenin and naringin in nanoliposomes based on the gradual reduction in their water solubility after the pH changed to acidity. The naringenin-loaded nanoliposomes were predominantly nanometric (44.95–104.4 nm), negatively charged (−14.1 to −19.3 mV) and exhibited relatively high encapsulation efficiency (EE = 95.34% for 0.75 mg/mL naringenin within 1% *w*/*v* lecithin). Additionally, the naringenin-loaded nanoliposomes still maintained good stability during 31 days of storage at 4 °C. Zhou et al. [2] fabricated a caseinate-stabilized thymol nanosuspension by pH-driven methods. Thymol was extremely stable at pH 7.0–12.0 even after incubation for 24 h, which means the loss of thymol during the pH-driven process is negligible. The physicochemical properties of thymol nanosuspensions are highly dependent on the caseinate concentration. Caseinate could stabilize thymol nanosuspensions even at a relatively low caseinate concentration, and the loading capacity can be as high as 45.9%. Chen et al. [3] compared two polysaccharides [sodium alginate (ALG) and sodium carboxymethyl cellulose (CMC)] to establish zein/sophorolipid/ALG (ALG/S/Z) and zein/sophorolipid/ALG (CMC/S/Z) nanoparticles to encapsulate 7,8-dihydroxyflavone (7,8-DHF), respectively. They found that CMC/S/Z possessed lower polydispersity index, particle size and turbidity, but higher zeta potential, encapsulation efficiency and loading capacity compared to ALG/S/Z. Compared to zein/sophorolipid nanoparticles (S/Z), both ALG/S/Z and CMC/S/Z had better stability against low pH (pH 3~4) and high ionic strengths (150~200 mM NaCl). Apart from the above particle forms, nanofibers have emerged as a novel delivery system due to its simplicity and effectiveness. Electrospinning, solution blow spinning, and eletro-blow spinning are the most common techniques for continuously producing nanofibers with a fiber diameter range from sub-nanometers to micrometers. The electrospun films were also adopted to stabilize the sensitive bioactives. Cui et al. [4] fabricated an antimicrobial food packaging film with controlled release by loading cinnamaldehyde (CIN) on etched halloysite nanotubes (T-HNTs) and adding it to sodium alginate (SA) matrix. It was found that CIN could be successfully loaded into the T-HNTs and the addition of T-HNTs-CIN significantly improved the water vapor barrier properties and tensile strength of the film. Additionally, the SA/T-HNTs-CIN film could delay the release of CIN into fatty food simulation solution compared with that of SA/CIN film. Wen et al. [5] reported that the incorporation of a Nervilia fordii extract (NFE) in the electrospun poly(vinyl alcohol) (PVA) and polyvinyl(pyrrolidone) (PVP) bio-composite film could retain its antioxidant capacity, avoiding the fish oil’s oxidation (and thus extending its shelf life). Yang et al. [6] fabricated gelatin/nylon 66 (PA66) composite nanofibers by solution blow spinning (SBS). Morphology observations show that GA/PA66 composite films had a nano-diameter from 172.3 to 322.1 nm. Nylon 66 (PA66) was proved to improve the mechanical properties and the ability to resist dissolution of gelatin nanofibrous films. Another study was performed by microfluidics to encapsulate lutein to improve its bioaccessibility in the gastrointestinal tract [7]. Two types of oils (safflower oil (SO) and olive oil (OL)) were selected as a delivery vehicle for lutein, and two customized microfluidic devices (co-flow and combination-flow) were used. The results demonstrated that the types of oil and device do not affect the lutein bioaccessibility. Findings from this study may provide scientific insights into emulsion-based delivery systems that employ microfluidics for the encapsulation of bioactive compounds into foods. Finally, Deng [8] systematically summarized that soy-based emulsifiers are currently extensively studied and applied in the food industry for its applications in bioactive and nutrient delivery.

Another focus of the published articles in this special issue is the sensitive detection of various contaminants (pesticides, drug, copper (II) ions, pathogenic bacterium) associated with food safety through different nano-techniques. In the study by Feng et al. [9], a novel nano/micro-structured pesticide detection card was developed by combining electrospinning and hydrophilic modification, and its feasibility for detecting different pesticides was investigated [9]. This self-made detection card showed a 5-fold, 2-fold, and 1.5-fold reduction of the minimum detectable concentration for carbofuran, malathion, and trichlorfon, respectively, compared to the national standard values. In another study, Li et al. [10] created a portable, rapid, and sensitive time-resolved fluorescence immunochromatography for on-site detection of dexamethasone in milk and pork. A parallel experiment for 20 milk and 10 pork samples with LC-MS/MS was carried out to confirm the performance of the developed TRFM-ICA. The results of the two methods are basically the same. In addition, the nanobody, as an important tool in immunoassay for chemical contaminants, was developed and its efficiency was examined by detecting a secondary metabolite of cyanobacteria, namely nodularin (NOD-R) [11]. The ic-ELISA method based on the nanobody N56 was validated with spiked water sample and confirmed by UPLC–MS/MS, which indicated that the ic-ELISA established in this work is a reproducible detection assay for nodularin residues in water samples. Xu et al. [12] prepared a highly sensitive and selective fluorescence probe that used mercaptopropionic acid (MPA)-capped InP/ZnS quantum dots (MPA-InP/ZnS QDs) for the detection of trace amounts of Cu^2+^ in water. This probe exhibited an extremely low limit of detection of 0.22 nM. Meanwhile, a possible fluorescence-quenching mechanism was proposed in this study. In another study, to achieve the rapid detection of *Listeria monocytogenes*, Zhu et al. [13] used aptamers for the original identification and built a photoelectrochemical aptamer sensor using exonuclease-assisted amplification. In brief, tungsten trioxide (WO_3_) was used as a photosensitive material, which was modified with gold nanoparticles to immobilize complementary DNA, and amplified the signal by means of the sensitization effect of CdTe quantum dots and the shearing effect of exonuclease I (Exo I) to achieve high-sensitivity detection. This strategy had a detection limit of 45 CFU/mL in the concentration range of 1.3 × 10^1^–1.3 × 10^7^ CFU/mL, providing a new way to detect *Listeria monocytogenes*.

Overall, these articles extend the knowledge on the application of nanomaterials in food nutrition and safety, promoting the development of nanotechnologies in food industry.

Finally, we would like to thank all of the authors for their submissions, and all of the referees for their valuable suggestions for improving the manuscripts.
